# De-novo design, synthesis and evaluation of novel 6,7-dimethoxy-1,2,3,4-tetrahydroisoquinoline derivatives as HIV-1 reverse transcriptase inhibitors

**DOI:** 10.1186/s13065-015-0111-6

**Published:** 2015-06-09

**Authors:** Subhash Chander, Penta Ashok, Anupam Singh, Sankaranarayanan Murugesan

**Affiliations:** Medicinal Chemistry Research Laboratory, Department of Pharmacy, Birla Institute of Technology & Science, Pilani, 333031 Rajasthan India

**Keywords:** Tetrahydroisoquinoline, HIV-1 RT, Docking, NNRTI, Glide, Resistance

## Abstract

**Background:**

Acquired Immune Deficiency Syndrome (AIDS) is the advanced stage of infection caused by Human Immunodeficiency Virus (HIV). HIV/AIDS had a great impact on society, both as an illness and as a source of discrimination. Non-Nucleoside Reverse Transcriptase Inhibitors (NNRTIs) are structurally diverse group of compounds which binds to Reverse Transcriptase (RT) enzyme of HIV. Like other anti-HIV drugs, long-term clinical effectiveness of approved NNRTIs has been hampered due to the rapid development of drug resistance. So, there is an urgent need to discover the NNRTIs, which can be effective against the drug sensitive as well as drug resistant strains of HIV-1 RT.

**Results:**

Two series of novel thirty, 6,7-dimethoxy-1,2,3,4-tetrahydroisoquinoline analogues (**5a-o**) and (**8a-o**) were designed and synthesized as inhibitor of HIV-1 reverse transcriptase. All the synthesized compounds were characterized by infrared spectroscopy, proton nuclear magnetic resonance spectroscopy, mass spectroscopy and evaluated for *in-vitro* RT inhibitory activity. Among the tested compounds, eighteen compounds exhibited more than 50 % inhibition at tested 100 μM concentration, in which two compounds **8h** and **8l** showed promising inhibition (74.82 and 72.58 %) respectively. The preliminary structure–activity relationship (SAR) of the test compounds and docking studies of the two significantly active compounds **8h** and **8l** were performed to examine their putative binding with HIV-RT. Predicted physiochemical parameters of the synthesized compounds were within the acceptable range of drugable properties.

**Conclusion:**

The results obtained from this investigation revealed that, the synthesized compounds (**5a-o**) and (**8a-o**) showed moderate to promising HIV-1 RT inhibition activity. The overall SAR studies can help in identification of further lead as well as in designing of newer potential inhibitor of HIV-1 RT.

**Graphical Abstract:**
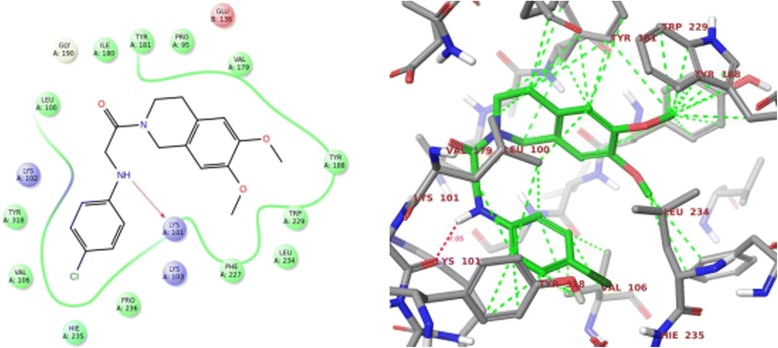
Best docked pose of compound **8h** inside the non-nucleoside inhibitory binding pocket of 3MEE enzyme.

## Background

**Fig. 1:**
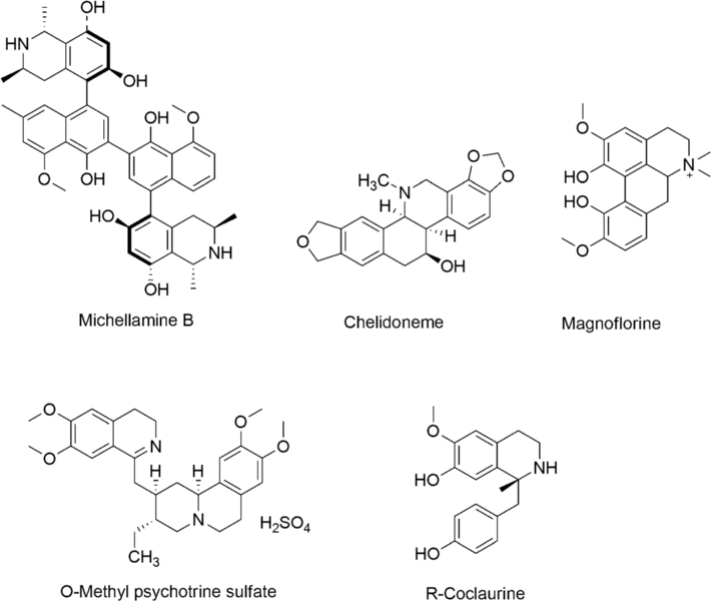
Natural THIQ derivatives reported as inhibitors of HIV-1 and target Reverse Transcriptase

Non-Nucleoside Reverse Transcriptase Inhibitors (NNRTIs) are important components of the preferred combination of antiretroviral therapy for the treatment of HIV infection [1, 2]. Tetrahydroisoquinoline (THIQ) scaffold containing natural products as well as synthetic derivatives have privileged position in the medicinal chemistry and reported for different biological activities like anti-microbial [3], anti-cancer [4], anti-viral [5], anti-HIV [6] and others [7, 8]. Natural THIQs as inhibitors of HIV-1 and its enzyme reverse transcriptase were continuously reported in the literature. For example, michellamine-B (Fig. 1) an alkaloid from *Ancistrocladus korupenis* was reported for anti-HIV activity [9]. Other THIQ derivatives (Fig. 1) reported in the literature against reverse transcriptase of HIV-1 were chelidoneme, magnoflorine [10], *O*-methyl psychotrine sulphate [11]. Another series of benzyl THIQ derivatives, isolated from the leaves of *Nelumbo nucifera* contains R-coclaurine (Fig. 1) as active constituent also showed potent anti-HIV activity [12].

**Fig. 2:**
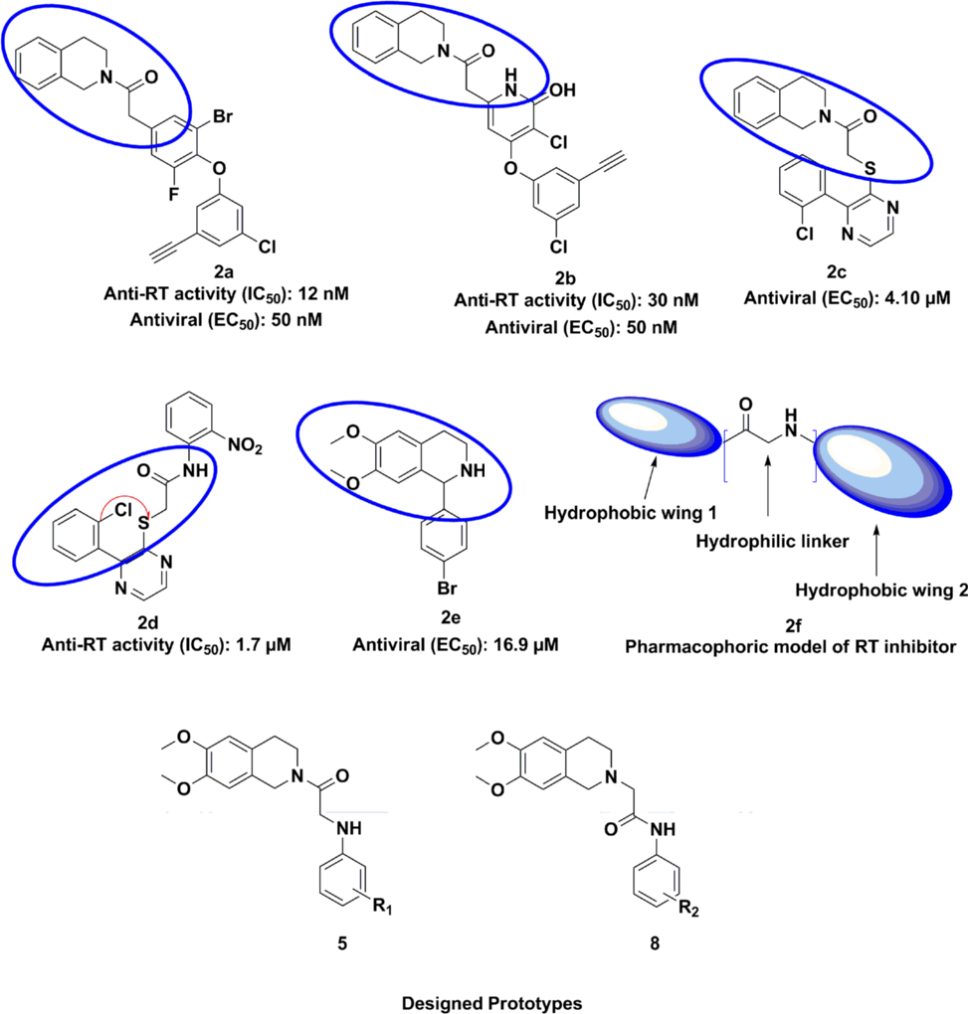
Structure of tetrahydroisoquinolines (**2a, 2b, 2c** and **2e**) and related analogue (**2d**) as potent inhibitor of HIV-1 and HIV-1 RT along with proposed pharmacophoric model (**2f**) and designed prototypes (**5** and **8**)

Literature study revealed that, apart from the THIQs obtained from the natural resources, their synthetic analogues also showed significant potency against HIV-1 RT. In a similar study, two novel derivatives of THIQ (Fig. 2a and b) showed excellent potency against wild strains of HIV-1 by inhibiting RT enzyme [13]. Another study [14] revealed that, compounds having pyrazine ring connected to the tetrahydroisoquinoline via thiaglycinamide linker (Fig. 2c) and its bioisosters (Fig. 2d)**,** exhibited good potency against HIV-1 RT with IC_50_ 4.10 and 1.7 μM respectively. In another study, a series of 1-aryl-6,7-dimethoxy-1,2,3,4-tetrahydroisoquinolines were synthesized and assayed for anti HIV-1 activity, most active compound of the series (Fig. 2e) showed good potency with EC_50_ 16.9 μM [6].

Even though, NNRTIs are structurally diverse compounds, still they contain numerous ubiquitous fragments in their structures and possess a common pharmacophoric model. This model includes an aromatic ring able to participate in *pi-pi* stacking interactions, amide or thio-amide moieties capable of hydrogen bonding and one or more hydrocarbon-rich domain that participate in hydrophobic interactions [15]. So considering these crucial pharmacophoric features of HIV-1 RT inhibitor, we generated a common pharmacophoric model (Fig. 2f). Based upon this model, new tetrahydroisoquinoline prototypes **5** and **8** were designed (Fig. 2). Further using these prototypes, two series of novel thirty compounds **5a-o** and **8a-o** were synthesized and evaluated for *in-vitro* RT inhibitory activity. Structure activity relationship (SAR) studies of the test compounds were investigated based upon the *in-vitro* RT inhibitory potency. Molecular docking studies of most active compound were also carried out in order to know exact binding pattern at the active site of the receptor. These studies may help in further lead identification and designing of more potent molecules against HIV-1 RT.

## Methods

### Chemistry

All solvents and reagents purchased from Sigma or Merck companies were used as received without further purification. Solvent system used throughout experimental work for running TLC was ethyl acetate and hexane mixture (in suitable proportion) in order to monitor the progress of reactions. Melting points were uncorrected and determined in open capillary tubes on a Precision Buchi B530 (Flawil, Switzerland) melting point apparatus containing silicon oil. IR spectra of the synthesized compounds were recorded using FTIR spectrophotometer (Shimadzu IR Prestige 21, India). ^1^H NMR spectra were recorded on a Bruker DPX-400 spectrometer (Bruker India Scientific Pvt. Ltd., Mumbai) using TMS as an internal standard (chemical shifts in *δ*). ESMS were recorded on MICROMASS Quattro-II LCMS system (Waters Corporation, Milford, USA).

### *In-vitro* HIV-1 RT inhibitory activity

Current study involved the use of enzymatic assay for *in-vitro* screening of compounds against HIV-1 RT, apart from this human being or other animals were not used in the study. Synthesised compounds were evaluated for *in-vitro* HIV-1 RT inhibitory potency using colorimetric assay method (Roche diagnostics) and carried out as described in the kit protocol. Marketed drug efavirenz was used as reference during the study. Test is based upon the colorimetric enzyme immunoassay, which quantitatively determines the retroviral reverse transcriptase activity. 100 μM concentrations of the test compounds were used for *in-vitro* assay. Briefly, the reaction mixture was set with template primer complex, RT enzyme and dNTPs in the lysis buffer with or without inhibitors. Reaction mixture was incubated at 37 °C for 1 h and then transferred to streptavidine-coated microtitre plate (MTP). The biotin-labeled dNTPs that are incorporated in the template due to activity of RT were bound to streptavidine. The unbound dNTPs were washed using wash buffer and anti-DIG-POD was added to the MTP. The DIG-labeled dNTPs incorporated in the template were bound to anti-DIG-POD antibody. The unbound anti-DIG-POD was washed and the peroxide substrate (ABST) was added to the MTP. A colored reaction product was produced during the cleavage of the substrate catalyzed by a peroxide enzyme. The absorbance of the sample was determined at optical density (OD) of 405 nm using micro titer plate ELISA reader.

The % inhibition of HIV-1 RT was calculated using the following formula:

$$ \%\ \mathrm{inhibition}=100-\left(\frac{\mathrm{OD}\ \mathrm{at}\ 405\ \mathrm{nm}\ \mathrm{with}\ \mathrm{inhibitor}}{\mathrm{OD}\ \mathrm{at}\ 405\ \mathrm{nm}\ \mathrm{with}\mathrm{out}\ \mathrm{inhibitor}}\times 100\right) $$

### Docking methodology

*In-silico* docking studies were performed using Glide 5.9 [16] running on maestro version 9.4, to investigate the exact binding mode of most active compound in NNIBP (Non-Nucleoside Inhibitory Binding Pocket) of HIV-1 RT [17]. Enzyme used for the docking study was wild RT, retrieved from RCSB Protein Data Bank (PDB id: 3MEE) in complex with rilpivirine. Protein preparation wizard of Schrödinger suite was used for preparation of retrieved 3MEE protein. Protein was pre-processed separately by deleting the substrate co-factor as well as the crystallographically observed water molecules (water without H bonds), followed by optimization of hydrogen bonds. After assigning charge and protonation state, finally energy was minimized with Root Mean Square Deviation (RMSD) value of 0.30 Å using Optimized Potentials for Liquid Simulations-2005 (OPLS-2005) force field [18]. Prepared protein and co-crystallized ligand was employed to build energy grids using the default value of protein atom scaling (1.0 Å) within a cubic box of dimensions, centered on the centroid of the X-ray ligand pose. The structures of the designed analogues were drawn using ChemSketch and converted to 3D structure with the help of 3D optimization tool. By using the LigPrep 2.6 [19] module, the drawn ligands were geometry optimized. Partial atomic charges were computed using OPLS-2005 force field. Finally, 32 poses were included with different tautomeric and steric features for docking studies. Finally, prepared ligands were docked with prepared protein using Glide 5.9 module, in extra precision mode (XP). The best docked pose (with lowest Glide score value) obtained from Glide was analyzed. RMSD value was calculated between the experimental binding mode of ligand rilpivirine as in X-ray co-crystallized pose in 3MEE and Glide re-docked pose to ensure accuracy and reliability of the docking procedure. Physicochemical parameters of the synthesized compounds were *in-silico* predicted in order to evaluate their drug likeness properties using Qik-prop 3.7 [20] module of Schrödinger and online tool AdmetSAR [21].

## Results and discussion

### Chemistry

**Scheme 1:**
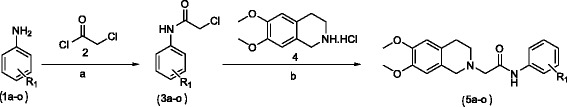
Synthesis of final compounds (**5a-o**), reagents and conditions: (**a**) DCM, Et_3_N, 0 °C-rt, 2 h; (**b**) ACN, K_2_CO_3_, Reflux 3–5 h

**Scheme 2:**
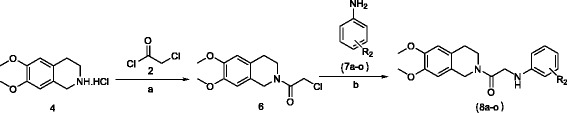
Synthesis of final compounds (**8a-o**), reagents and conditions: (**a**) DCM, Et_3_N, 0 °C-rt, 2.5 h; (**b**) neat, K_2_CO_3_, microwave irradiation at 300 W for 2 to 4.5 min

The synthesis of target compounds **5a-o** and **8a-o** is outlined in Schemes 1 and 2 respectively. In Scheme 1, reaction of substituted anilines **1a-o** and chloroacetyl chloride **2** in DCM, in the presence of triethylamine as a base afforded substituted 2-chloro-*N*-phenylacetamide (**3a-o),** which on subsequent treatment with hydrochloride salt of 6,7-dimethoxy tetrahydroisoquinoline (**4**), in acetonitrile in the presence of potassium carbonate as base afforded the title compounds (**5a-o**). In Scheme 2, reaction of chloroacetyl chloride (**2**) and 6,7-dimethoxy tetrahydroisoquinoline (**4**) in DCM as solvent and triethylamine as base gave intermediate **6**. Further, for the synthesis of final compounds of series **8**, reaction conditions were standardized using the conventional as well as microwave assisted synthetic approaches. Finally reaction of intermediate **6** with substituted anilines (**7a-o)** under solvent free, microwave condition in the presence of potassium carbonate as base afforded the final compounds in comparatively better yield than the conventional synthetic approach **(8a-o).**

All the synthesized compounds were characterized by spectral analysis. The IR spectra of **5a-o** showed the expected absorption bands of amide hydrogen (−CO**NH**-) at the region 3200–3500 cm^−1^ (strong, broad). Likewise compounds **8a-o** contained secondary nitrogen (−N**H**-) which showed absorption peak in the region 3250–3400 cm^−1^ (strong, sharp). All the synthesized compounds possessed characteristic amide carbonyl (**C = O)** peak at 1630–1690 cm^−1^ in its expected region. ^1^H NMR signals and proton counting of the title compounds were also observed in their expected region. ESI-MS of the synthesized compounds showed the corresponding M + 1 peak.

### *In-vitro* HIV-1 RT screening

*In-vitro* studies of the test compounds (**5a-o**), showed weak to moderate activity against HIV-1 RT (Table 1). Among the tested compounds, five compounds (**5d, 5f, 5h, 5n** and **5o**) showed more than 50 % enzyme inhibition at tested 100 μM concentration. Substitutions with electron donating groups especially at the *para* position of the phenyl ring enhanced the potency against HIV-1 RT, for example *p*-methyl substituted compound **5d** (52.46 % inhibition) exhibited 1.5 times more potent as compared to un-substituted one (**5a**) (34.32 % inhibition). Similarly, *p*-methoxy substituted compound **5f** is more potent (56.23 % inhibition) as compared to the *m*-substituted compound **5e** (44.21 % inhibition). Substitution with electron withdrawing group also altered potency against HIV-1 RT, halogen groups like chloro substituted compounds (**5h, 5i** and **5j**) followed the pattern in decreasing order of potency *ortho* > *meta* > *para*. Similarly compound **5g** having *p-*fluoro substitution decreased the potency, while substitution of phenyl ring with *p-*nitro group (**5k**) slightly enhanced the potency. Compound **5l** with strong electron withdrawing group (*m*-trifluoromethyl) also showed less potency against HIV-1 RT. Upon di-substitution at phenyl ring, good enhancement in the potency was observed (compounds **5m, 5n** and **5o**). Compound **5n** (3,4-dimethyl substitution) showed highest potency (58.12 % inhibition) among the tested **5a-o** series of compounds.

**Table 1:** *In-vitro* HIV-1 RT inhibition results of the test compounds

Compound code	R	% RT Inhibition^a^	Compound code	R	% RT Inhibition^a^
5a	H	34.32	8a	2-OCH_3_	45.31
5b	2-CH_3_	36.23	8b	3-OCH_3_	51.32
5c	3-CH_3_	39.51	8c	4-OCH_3_	57.45
5d	4-CH_3_	52.46	8d	3-F	48.37
5e	3-OCH_3_	44.21	8e	4-F	53.93
5f	4-OCH_3_	56.23	8f	2-Cl	61.38
5g	4-F	28.45	8g	3-Cl	63.74
5h	2-Cl	52.34	8h	4-Cl	74.82
5i	3-Cl	37.26	8i	2-Br	63.38
5j	4-Cl	33.65	8j	3-Br	60.46
5k	4-NO_2_	42.78	8k	4-Br	68.63
5l	3-CF_3_	30.64	8l	4-CN	72.58
5m	2,6-dimethyl	48.36	8m	3-Aceto	54.75
5n	3,4-dimethyl	58.12	8n	3-CF_3_	66.74
5o	2-CH_3_-5-Cl	53.76	8o	2,5 dimethyl	63.64
Efavirenz		98.12			

^a^Data are indicated as percentage of inhibition of HIV-1 RT at 100 μM concentration

Majority of compounds in **8a-o series** showed more than 50 % inhibition of HIV-1 RT at 100 μM tested concentration except compounds **8a** and **8d** (Table 1). Substitutions with electron-donating groups at phenyl ring like methoxy (compounds **8a, 8b** and **8c)** showed moderate potency against RT. Compounds substituted with electron withdrawing groups like fluoro (**8d** and **8e**), potency was not changed much may be due to its very small size and very strong electron withdrawing nature. However substitution with other electron withdrawing groups like chloro, bromo, nitro and aceto (**8f, 8g, 8h, 8i, 8j, 8k, 8l, 8m** and **8n**) on the phenyl ring increased the potency. Compounds having chloro substitution at the *ortho* and *meta* position (**8f** and **8g**) did not showed any significant difference in potency, but substitution at *para* position (**8h**) significantly enhanced the potency (74.82 % inhibition of HIV-RT). Like chloro substituted compounds almost similar pattern of potency was exhibited by the bromo substituted compounds (**8i, 8j and 8k)**. Substitution with cyano, an electron withdrawing group at the *para* position (**8l**) also significantly increased the potency. Apart from this, compound **8m** and **8n** having aceto and trifluoromethyl group at the *meta* position showed moderate to good potency (54.75 and 66.74 % inhibition) respectively against RT. Further, dimethyl substituted compound (**8o**) found to be more potent as compared to the single methoxy substituted compounds (**8a, 8b** and **8c**). Among all the tested compounds, analogue (**8h**) exhibited highest potency (74.82 % inhibition of HIV-1 RT).

### Docking studies

**Fig. 3:**
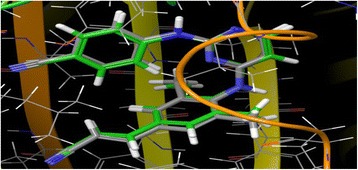
Re-docked pose of rilpivirine (green) with X-ray co-crystallized (gray) structure in NNIBP of 3MEE

In order to predict the exact binding mode and to know the interactions of most active compounds (**8h** and **8l**) within NNIBP of HIV-1 RT, docking studies were carried out on X-ray coordinates of HIV-1 RT enzyme (PDB ID: 3MEE). To evaluate the accuracy and reliability of the docking procedure, co-crystallized native ligand rilpivirine was removed from its binding site of 3MEE and again subjected to dock into the same binding pocket (Fig. 3). As a result, the value of RMSD obtained between experimental binding mode as in X-ray and re-docked pose for rilpivirine was 0.8, which suggested that docking procedure could be relied onto predict the exact binding mode of the designed compounds.

**Fig. 4:**
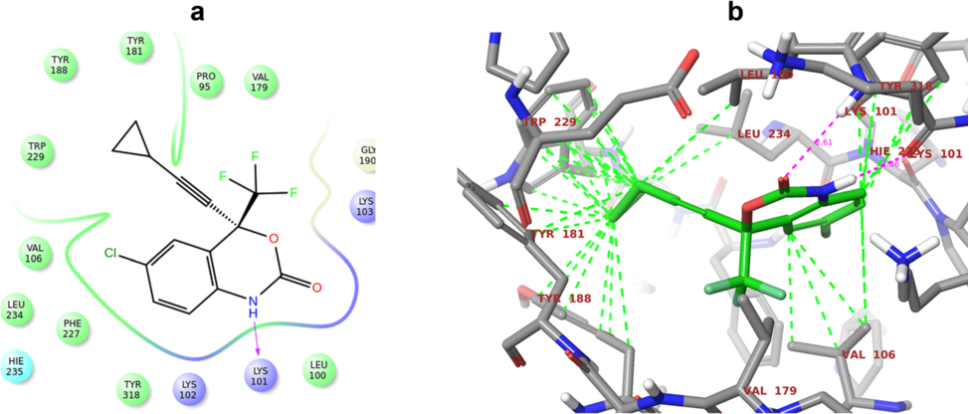
Docking poses of efavirenz at NNIBP of RT enzyme, showing two dimensional interactive diagram (**a**), hydrophobic and hydrogen bond interaction (**b**) represented by green and pink dotted lines respectively

Docking studies of the reference drug efavirenz showed that, its cyclopropyl ring extents towards deep hydrophobic pocket surrounded by amino acids Tyr-188, Tyr-181, Leu-100 and Trp-229 and chloro phenyl ring showed hydrophobic interaction with Tyr-318 and Val-106 residues (Fig. 4). Apart from hydrophobic interaction, NH of the efavirenz form strong hydrogen bond interaction with the C = O of the Lys-101. These strong hydrophobic and hydrophilic interactions of efavirenz with receptor may accounts for good *in-silico* activity.

**Fig. 5:**
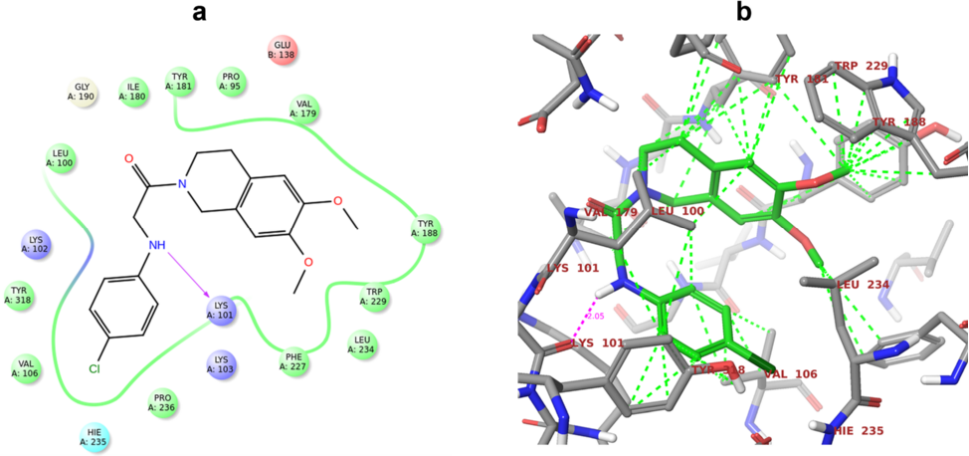
Docked pose of **8h** at NNIBP of RT enzyme, showing two dimensional interactive diagram (**a**), hydrophobic and hydrogen bond interaction (**b**) represented by green and pink dotted lines respectively

**Fig. 6:**
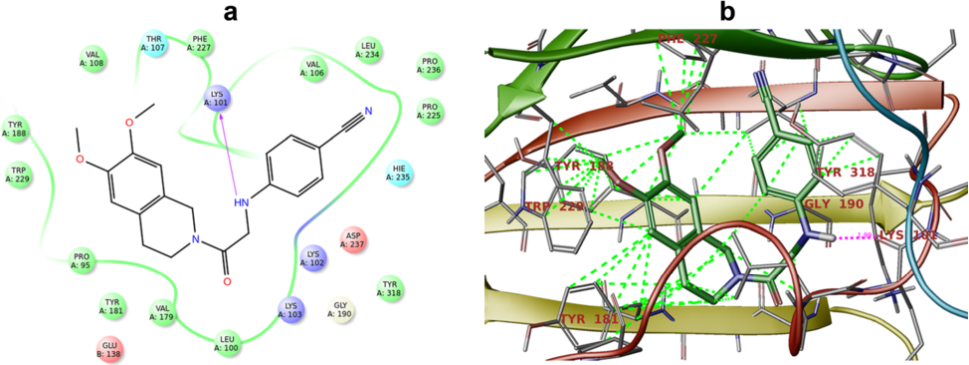
Docked pose of **8l** at NNIBP of RT enzyme, showing two dimensional interactive diagram (**a**), hydrophobic and hydrogen bond interaction (**b**) represented by green and pink dotted lines respectively

However, analysis of best docked pose of two most active compounds **8h** and **8l** revealed that their 6,7-dimethoxytetrahydroisoquinoline nucleus form strong hydrophobic interaction with amino acids like Tyr-188, Tyr-181 and Trp-229 (Figs. 5 and 6). Other hydrophobic wing of **8h** (*p-*chloro phenyl) showed hydrophobic interactions with Val-106 and Tyr-318 (Fig. 5b), while second wing of **8h** (*p-*cyano phenyl) exhibited hydrophobic interactions predominantly with Tyr- 318 (Fig. 6b). Moreover NH of both ligands form hydrogen bond interaction with Lys-101, which is generally considered crucial for the RT inhibition activity and additionally enhanced the binding affinity of ligands **8h** and **8l** with HIV-1 RT. Substitution at the *para* position of phenyl especially with electron withdrawing groups of moderate size like chloro and cyano in **8h** and **8l** respectively enhanced the hydrophobic area of contact between the ligand-receptor complex and may be responsible for their significant *in-vitro* activity.

### Predicted *in-silico* drug likeness properties

ADMET (Absorption, Distribution, Metabolism, Excretion and Toxicological) parameters play very crucial role in the discovery and development of novel drugs. Currently available drugs in the market possess a balance of desirable ADMET properties and intrinsic potency. Lipinski's rule of five (RO5) helps to predict the presence of drug-like physicochemical properties in a given compound, these properties affect as drug’s pharmacokinetics (ADMET) in the human body. Drug candidates that comply with the Lipinski rule of five have less failure rate during the clinical trial.

So in our study, physicochemical parameters of the synthesized compounds were *in-silico* generated in order to evaluate their drug likeness properties using Qik-prop module of Schrödinger and online tool AdmetSAR (Table 2). Results obtained in this study revealed that, the *in-silico* generated parameters of all synthesized derivatives were within the acceptable range of drug-likeness. General range of physicochemical parameters for drug likeness varies as: Mol. wt. (150–650), H bond donor (≤5), H bond acceptor (≤10) Log Po/w (−0.4 to 5.6), Log S (−6.5 to −0.5), Log BB (−3.0 to 1.2), % Human oral absorption (≥80 % is high and ≤25 % is poor).

**Table 2:** *In-silico* predicted physiochemical parameters of the test compounds

Compound code	Mol. wt.	Mol. Vol.	H bond donor	H bond acceptor	Log Po/w	Log S	Log BB	Human oral absorption (%)
5a	326.39	1099.45	1	5	3.12	−3.62	0.19	98.23
5b	340.42	1135.79	1	5	3.3	−3.75	0.23	97.35
5c	340.42	1160.54	1	5	3.4	−4.21	0.17	96.65
5d	340.42	1160.72	1	5	3.43	−4.21	0.17	97.78
5e	356.42	1174.76	1	6	3.21	−3.83	0.11	97.21
5f	356.42	1175.85	1	6	3.21	−3.85	0.11	95.16
5g	344.38	1116.84	1	5	3.36	−4.01	0.30	96.45
5h	360.83	1137.53	1	5	3.59	−4.18	0.40	97.32
5i	360.83	1144.61	1	5	3.62	−4.36	0.37	96.15
5j	360.83	1144.83	1	5	3.62	−4.38	0.35	95.68
5k	371.39	1173.43	2	6	2.40	−3.78	−0.91	78.41
5l	394.39	1199.11	1	5	4.11	−5.08	0.45	94.32
5m	354.44	1187.59	1	5	3.66	−4.14	0.30	95.41
5n	354.44	1211.12	1	5	3.70	−4.62	0.17	97.16
5o	374.86	1194.24	1	5	3.92	−4.63	0.44	98.24
8a	356.42	1171.02	1	6	3.27	−4.02	−0.33	95.55
8b	356.42	1169.84	1	6	3.22	−4.13	−0.38	96.36
8c	356.42	1170.15	1	6	3.22	−4.14	−0.38	94.64
8d	344.38	1111.12	1	5	3.37	−4.29	−0.20	97.80
8e	344.38	1111.09	1	5	3.37	−4.29	−0.20	96.45
8f	360.83	1130.02	1	5	3.53	−4.51	−0.16	98.12
8g	360.83	1139.08	1	5	3.62	−4.66	−0.14	97.86
8h	360.83	1139.13	1	5	3.62	−4.66	−0.14	98.64
8i	405.29	1137.62	1	5	3.60	−4.61	−0.14	99.16
8j	405.29	1147.99	1	5	3.70	−4.77	−0.13	98.20
8k	405.29	1148.04	1	5	3.70	−4.77	−0.13	97.56
8l	351.40	1160.41	1	6	2.37	−4.87	−1.14	86.13
8m	368.43	1216.51	1	6	2.60	−4.12	−0.95	90.86
8n	394.39	1193.18	1	5	4.11	−5.36	−0.04	98.54
8o	354.44	1209.19	1	5	3.80	−5.05	−0.22	99.22
Efavirenz	315.67	880.03	1	3	3.52	−4.86	0.10	94

## Experimental

### General procedure for the synthesis of substituted 2-chloro-*N*-phenylacetamide (3a-o)

Chloroacetyl chloride **2** (1.68 g, 1.5 mmol) was added dropwise to the ice cooled stirring solution of substituted anilines (**1a-o)** (1.5 mmol) in DCM, in the presence of triethyl amine (4.5 mmol, 0.46 g) as base. Reaction was further stirred at rt for 2 to 3 h, progress of the reaction was monitored with TLC. After completion of reaction, DMC layer was washed with distilled water, dried over sodium sulphate and evaporated on rotary evaporator to yield the intermediates (**3a-o)**.

### General procedure for the synthesis of substituted 2-(6,7-dimethoxy-3,4-dihydroisoquinolin-2(*1H*)-yl)-*N*-phenyl acetamide (5a-o)

Substituted 2-chloro-*N*-phenylacetamide (intermediates **3a-o,** 1 mmol) was added to the stirring reaction mass of **4**, in acetonitrile in the presence of potassium carbonate as base**.** Reaction mass was further allowed to reflux for 5 to 7 h, progress of the reaction was monitored by the TLC (Scheme 1). After completion of reaction, solvent acetonitrile was evaporated on rotary evaporator; water was added to the reaction mixture and extracted twice with equal volume (25 ml) of ethyl acetate. Combined ethyl acetate layer was washed with brine water, dried over sodium sulphate and evaporated on rotary evaporator to afford the final compounds **5a-o.**

### Synthesis of 2-chloro-1-(3,4-dihydro-6,7-dimethoxyisoquinolin-2(1H)-yl)ethanone (6)

Chloroacetyl chloride **2** (2.24 g, 20 mmol) was added dropwise to the ice cooled stirring solution of starting material **4** (4.6 g, 20 mmol) in DCM, in a round-bottom flask using triethyl amine (7.0 ml, 50 mmol,) as base (Scheme 2). Reaction mixture was further stirred at rt for 2.5 h until completion as per TLC. After completion of reaction, DMC layer was washed with distilled water once and then with brine water and dried over sodium sulphate and finally evaporated on rotary evaporator to afford the titled compounds.

### General procedure for the synthesis of substituted 1-(6,7-dimethoxy-3,4-dihydroisoquinolin-2(*1H*)-yl)-2-(phenylamino) ethanone using Microwave irradiation (8a-o)

2-methoxyaniline (**7a**) (0.12 g, 1 mmol) and chloro intermediate **6** (0.27 g, 1 mmol) was taken in microwave reaction vial in presence of potassium carbonate as base (0.347 g, 2.5 mmol) (Scheme 2). The microwave oven model Cata RI was programmed to 300 W at 100 °C for 2 to 4.5 min. The reaction was monitored using TLC. After completion of reaction, ice water was added to the reaction mixture which resulted the precipitation of the product. The solid product was filtered off and washed with excess cold water and dried to afford product **8a**. Similar apporoach was followed for the synthesis of rest of the compounds (**8b-o**).

### The spectral characterization of the synthesized derivatives

#### *2-(6,7-dimethoxy-3,4-dihydroisoquinolin-2(1H)-yl)-N-phenylacetamide* (5a)

Yield 73 %; white solid, MF: C_19_H_22_N_2_O_3_; MW: 326.16; MP: 151–154 °C; IR (KBr, ν, cm^−1^): 3288, 3001, 2821, 2775, 1693, 1517, 1423, 1228, 1139, 1105; ^1^H NMR (400 MHz, CDCl_3_) δ 9.21 (s, 1H), 7.59 (dd, *J* = 8.6, 1.1 Hz, 2H), 7.39 – 7.31 (m, 2H), 7.14 (d, *J* = 7.4 Hz, 1H), 6.67 (s, 1H), 6.55 (s, 1H), 3.88 (d, *J* = 12.2 Hz, 6H), 3.77 (s, 2H), 3.34 (s, 2H), 2.92 (s, 4H); MS: m/z 327 (M + 1).

#### *2-(6,7-dimethoxy-3,4-dihydroisoquinolin-2(1H)-yl)-N-o-tolylacetamide* (5b)

Yield 76 %; white solid, MF: C_20_H_24_N_2_O_3_; MW: 340.18; MP: 137–139 °C; IR (KBr, ν, cm^−1^): 3294, 2975, 2914, 2829, 1670, 1490, 1251, 1226, 1139, 1013; ^1^H NMR (400 MHz, CDCl_3_) δ 9.33 (s, 1H), 8.14 (d, *J* = 8.1 Hz, 1H), 7.25 (s, 1H), 7.17 (d, *J* = 7.2 Hz, 1H), 7.06 (td, *J* = 7.5, 1.2 Hz, 1H), 6.66 (s, 1H), 6.54 (s, 1H), 3.89 (s, 3H), 3.86 (s, 3H), 3.79 (s, 2H), 3.38 (s, 2H), 2.95 (d, *J* = 3.8 Hz, 4H), 2.20 (s, 3H); MS: m/z 341 (M + 1).

#### *2-(6,7-dimethoxy-3,4-dihydroisoquinolin-2(1H)-yl)-N-m-tolylacetamide* (5c)

Yield 78 %; white solid, MF: C_20_H_24_N_2_O_3_; MW: 340.18; MP: 131–133 °C; IR (KBr, ν, cm^−1^): 3286, 3010, 2949, 2833, 1683, 1519, 1489, 1220, 1143, 1024; 1H NMR (400 MHz, CDCl3) δ 9.17 (s, 1H), 7.40 (d, J = 11.8 Hz, 2H), 7.23 (t, J = 7.7 Hz, 1H), 6.95 (d, J = 7.6 Hz, 1H), 6.67 (s, 1H), 6.55 (s, 1H), 3.88 (d, J = 12.3 Hz, 6H), 3.74 (d, J = 17.9 Hz, 2H), 3.33 (s, 2H), 2.98 – 2.80 (m, 4H), 2.36 (s, 3H); MS: m/z 341 (M + 1).

#### *2-(6,7-dimethoxy-3,4-dihydroisoquinolin-2(1H)-yl)-N-p-tolylacetamide* (5d)

Yield 81 %; white solid, MF: C_20_H_24_N_2_O_3_; MW: 340.18; MP: 151–153 °C; IR (KBr, ν, cm^−1^): 3267, 2943, 2827, 2779, 1685, 1517, 1462, 1253, 1196; ^1^H NMR (400 MHz, CDCl_3_) δ 9.14 (s, 1H), 7.51 – 7.42 (m, 2H), 7.15 (d, *J* = 8.2 Hz, 2H), 6.67 (s, 1H), 6.55 (s, 1H), 3.88 (d, *J* = 12.1 Hz, 6H), 3.76 (s, 2H), 3.32 (s, 2H), 2.91 (d, *J* = 3.1 Hz, 3H), 2.33 (s, 3H); MS: m/z 341 (M + 1).

#### *2-(6,7-dimethoxy-3,4-dihydroisoquinolin-2(1H)-yl)-N-(3-methoxyphenyl)acetamide* (5e)

Yield 76 %; white solid, MF: C_20_H_24_N_2_O_4_; MW: 356.18; MP: 139–141 °C; IR (KBr, ν, cm^−1^): 3277, 2922, 2829, 1685, 1597, 1523, 1465, 1255, 1139, 1005; 1H NMR (400 MHz, CDCl3) δ 9.17 (s, 1H), 7.42 (d, J = 11.7 Hz, 2H), 7.24 (t, J = 7.8 Hz, 1H), 6.93 (d, J = 7.8 Hz, 1H), 6.66 (s, 1H), 6.54 (s, 1H), 3.88 (d, J = 12.3 Hz, 6H), 3.82 (s, 3H), 3.72 (d, J = 17.9 Hz, 2H), 3.32 (s, 2H), 2.96 – 2.78 (m, 4H); MS: m/z 357 (M + 1).

#### *2-(6,7-dimethoxy-3,4-dihydroisoquinolin-2(1H)-yl)-N-(4-methoxyphenyl)acetamide* (5f)

Yield 84 %; white solid, MF: C_20_H_24_N_2_O_4_; MW: 356.18; MP: 153–155 °C; IR (KBr, ν, cm^−1^): 3317, 2957, 2920, 2821, 1688, 1615, 1517, 1467, 1253, 1134, 1103; ^1^H NMR (400 MHz, CDCl_3_) δ 9.09 (s, 1H), 7.51 (s, 1H), 7.48 (s, 1H), 6.89 (d, *J* = 2.2 Hz, 1H), 6.87 (d, *J* = 2.2 Hz, 1H), 6.67 (s, 1H), 6.55 (s, 1H), 3.90 (s, 3H), 3.87 (s, 3H), 3.81 (s, 3H), 3.76 (s, 2H), 3.32 (s, 2H), 2.92 (s, 4H); MS: m/z 357 (M + 1).

#### *2-(6,7-dimethoxy-3,4-dihydroisoquinolin-2(1H)-yl)-N-(4-fluorophenyl)acetamide* (5g)

Yield 71 %; white solid, MF: C_20_H_24_N_2_O_4_; MW: 344.15; MP: 153–155 °C; IR (KBr, ν, cm^−1^): 3302, 3070, 2991, 2816, 1687, 1612, 1508, 1469, 1228, 1136, 1101; ^1^H NMR (400 MHz, CDCl_3_) δ 9.20 (s, 1H), 7.65 – 7.49 (m, 2H), 7.08 – 7.00 (m, 2H), 6.67 (s, 1H), 6.55 (s, 1H), 3.88 (d, *J* = 11.6 Hz, 6H), 3.76 (s, 2H), 3.33 (s, 2H), 2.92 (s, 4H), 1.60 (s, 2H); MS: m/z 345 (M + 1).

#### *N-(2-chlorophenyl)-2-(6,7-dimethoxy-3,4-dihydroisoquinolin-2(1H)-yl)acetamide* (5h)

Yield 64 %; white solid, MF: C_19_H_21_ClN_2_O_3_; MW: 360.12; MP: 109–111 °C; IR (KBr, ν, cm^−1^): 3298, 2993, 2889, 2833, 1701, 1519, 1436, 1230, 1139, 1103; ^1^H NMR (400 MHz, CDCl_3_) δ 9.97 (s, 1H), 8.48 (dd, *J* = 8.3, 1.5 Hz, 1H), 7.37 (dd, *J* = 8.0, 1.4 Hz, 1H), 7.32 (dd, *J* = 7.5, 1.0 Hz, 1H), 7.10 – 7.01 (m, 1H), 6.67 (s, 1H), 6.55 (s, 1H), 3.90 (s, 3H), 3.86 (s, 3H), 3.81 (s, 2H), 3.38 (s, 2H), 2.94 (s, 4H); MS: m/z 361 (M + 1).

#### *N-(3-chlorophenyl)-2-(6,7-dimethoxy-3,4-dihydroisoquinolin-2(1H)-yl)acetamide* (5i)

Yield 69 %; white solid, MF: C_19_H_21_ClN_2_O_3_; MW: 360.12; MP: 176–178 °C; IR (KBr, ν, cm^−1^): 3228, 3001, 2821, 2752, 1663, 1517, 1423, 1228, 1138, 1005; 1H NMR (400 MHz, CDCl3) δ 9.27 (s, 1H), 7.69 (t, J = 2.0 Hz, 1H), 7.45 (ddd, J = 8.2, 2.0, 0.9 Hz, 1H), 7.10 (ddd, J = 8.0, 2.0, 1.0 Hz, 1H), 6.67 (s, 1H), 6.55 (s, 1H), 3.89 (d, J = 12.4 Hz, 6H), 3.76 (s, 2H), 3.33 (s, 2H), 2.92 (d, J = 3.5 Hz, 4H); MS: m/z 361 (M + 1).

#### *N-(4-chlorophenyl)-2-(6,7-dimethoxy-3,4-dihydroisoquinolin-2(1H)-yl)acetamide* (5j)

Yield 74 %; white solid, MF: C_19_H_21_ClN_2_O_3_; MW: 360.12; MP: 177–179 °C; IR (KBr, ν, cm^−1^): 3269, 2941, 2900, 2825, 2785, 1680, 1517, 1492, 1396, 1253, 1220, 1141, 1105; ^1^H NMR (400 MHz, CDCl_3_) δ 9.25 (s, 1H), 7.56 – 7.53 (m, 2H), 7.31 (s, 2H), 6.67 (s, 1H), 6.55 (s, 1H), 3.90 (s, 3H), 3.87 (s, 3H), 3.76 (s, 2H), 3.33 (s, 2H), 2.92 (s, 4H); MS: m/z 361 (M + 1).

#### *2-(6,7-dimethoxy-3,4-dihydroisoquinolin-2(1H)-yl)-N-(4-nitrophenyl)acetamide* (5k)

Yield 67 %; light yellow solid, MF: C_19_H_21_N_3_O_5_; MW: 371.15; MP: 192–194 °C; IR (KBr, ν, cm^−1^): 3273, 2933, 2829, 1703, 1504, 1336, 1226; ^1^H NMR (400 MHz, CDCl_3_) δ 9.62 (s, 1H), 8.23 (d, *J* = 9.2 Hz, 2H), 7.77 (d, *J* = 9.2 Hz, 2H), 6.68 (s, 1H), 6.55 (s, 1H), 3.89 (d, *J* = 14.3 Hz, 6H), 3.78 (s, 2H), 3.38 (s, 2H), 2.94 (s, 4H); MS: m/z 372 (M + 1).

#### *2-(6,7-dimethoxy-3,4-dihydroisoquinolin-2(1H)-yl)-N-(3-(trifluoromethyl)phenyl) acetamide* (5l)

Yield 68 %; white solid, MF: C_20_H_21_F_3_N_2_O_3_; MW: 394.15; MP: 176–178 °C; IR (KBr, ν, cm^−1^): 3302, 3070, 2991, 2816, 1687, 1612, 1508, 1469, 1228, 1136, 1101; ^1^H NMR (400 MHz, CDCl_3_) δ 9.54 (s, 1H), 7.90 (s, 1H), 7.77 (d, *J* = 7.9 Hz, 1H), 7.45 (t, *J* = 7.9 Hz, 1H), 7.36 (d, *J* = 7.7 Hz, 1H), 6.68 (s, 1H), 6.58 (s, 1H), 3.90 (s, 3H), 3.86 (d, *J* = 9.9 Hz, 5H), 3.73 (d, *J* = 14.1 Hz, 1H), 3.46 (d, *J* = 6.7 Hz, 1H), 2.91 (d, *J* = 5.3 Hz, 2H), 2.88 – 2.76 (m, 2H); MS: m/z 395 (M + 1).

#### *2-(6,7-dimethoxy-3,4-dihydroisoquinolin-2(1H)-yl)-N-(2,6-dimethylphenyl)acetamide* (5m)

Yield 77 %; white solid, MF: C_21_H_26_N_2_O_3_; MW: 354.19; MP: 155–157 °C; IR (KBr, ν, cm^−1^): 3315, 2956, 2920, 2821, 1691, 1610, 1517, 1467, 1253, 1132, 1103; ^1^H NMR (400 MHz, CDCl_3_) δ 8.76 (s, 1H), 7.10 (d, *J* = 1.6 Hz, 3H), 6.66 (s, 1H), 6.56 (s, 1H), 3.88 (d, *J* = 3.5 Hz, 6H), 3.83 (s, 2H), 3.40 (s, 2H), 2.99 (d, *J* = 5.0 Hz, 2H), 2.95 (d, *J* = 5.2 Hz, 2H), 2.26 (s, 6H); MS: m/z 355 (M + 1).

#### *2-(6,7-dimethoxy-3,4-dihydroisoquinolin-2(1H)-yl)-N-(3,4-dimethylphenyl)acetamide* (5n)

Yield 75 %; white solid, MF: C_21_H_26_N_2_O_3_; MW: 354.19; MP: 158–160 °C; IR (KBr, ν, cm^−1^): 3332, 3045, 2997, 2831, 2773, 1693, 1519, 1489, 1225, 1230, 1138, 1101; ^1^H NMR (400 MHz, CDCl_3_) δ 9.11 (s, 1H), 7.35 (s, 2H), 7.09 (d, *J* = 7.9 Hz, 1H), 6.67 (s, 1H), 6.55 (s, 1H), 3.90 (s, 3H), 3.87 (s, 3H), 3.76 (s, 2H), 3.33 (s, 2H), 2.92 (s, 4H), 2.25 (d, *J* = 9.1 Hz, 6H); MS: m/z 355 (M + 1).

#### *N-(5-chloro-2-methylphenyl)-2-(6,7-dimethoxy-3,4-dihydroisoquinolin-2(1H)-yl) acetamide* (5o)

Yield 68 %; white solid, MF: C_20_H_23_ClN_2_O_3_; MW: 374.14; MP: 117–119 °C; IR (KBr, ν, cm^−1^): 3277, 3016, 2937, 2900, 2829, 1701, 1514, 1445, 1259, 1228, 1190, 1136; 1H NMR (400 MHz, CDCl3) δ 9.44 (s, 1H), 8.02 (dd, J = 6.7, 2.7 Hz, 1H), 7.22 – 7.12 (m, 2H), 6.67 (s, 1H), 6.54 (s, 1H), 3.88 (d, J = 11.5 Hz, 6H), 3.80 (s, 2H), 3.38 (s, 2H), 3.04 – 2.86 (m, 4H), 2.25 (s, 3H); MS: m/z 375 (M + 1).

#### *1-(6,7-dimethoxy-3,4-dihydroisoquinolin-2(1H)-yl)-2-(2-methoxyphenylamino) ethanone* (8a)

Yield 73 %; white solid, MF: C_20_H_24_N_2_O_4_; MW: 356.17; MP: 91–92 °C; IR (KBr, ν, cm^−1^): 3419, 3062, 2991, 2833, 1656, 1604, 1514, 1415, 1263, 1234, 1118; ^1^H NMR (400 MHz, CDCl_3_) δ 7.32 (dd, *J* = 9.4, 4.5 Hz, 1H), 7.12 (s, 1H), 6.69 – 6.64 (m, 2H), 6.64 – 6.53 (m, 2H), 4.76 (s, 1H), 4.58 (s, 1H), 4.06 (s, 1H), 3.93 (t, *J* = 5.9 Hz, 1H), 3.91 – 3.83 (m, 7H), 3.76 (s, 3H), 3.70 (t, *J* = 5.9 Hz, 1H), 2.92 (t, *J* = 5.7 Hz, 1H), 2.86 (t, *J* = 5.7 Hz, 1H); MS: m/z 257 (M + 1).

#### *1-(6,7-dimethoxy-3,4-dihydroisoquinolin-2(1H)-yl)-2-(3-methoxyphenylamino) ethanone* (8b)

Yield 79 %; white solid, MF: C_20_H_24_N_2_O_4_; MW: 356.17; MP: 118–119 °C; IR (KBr, ν, cm^−1^): 3361, 2999, 2953, 2833, 1643, 1606, 1517, 1421, 1224, 1163, 1112; ^1^H NMR (400 MHz, CDCl_3_) δ 7.13 (t, *J* = 8.1 Hz, 1H), 6.74 – 6.60 (m, 2H), 6.32 (dt, *J* = 5.8, 3.0 Hz, 2H), 6.22 (d, *J* = 1.7 Hz, 1H), 4.74 (s, 1H), 4.56 (s, 1H), 3.97 (s, 2H), 3.94 – 3.86 (m, 7H), 3.81 (s, 3H), 3.68 (t, *J* = 5.9 Hz, 1H), 2.89 (t, *J* = 5.9 Hz, 1H), 2.83 (t, *J* = 5.9 Hz, 1H); MS: m/z 257 (M + 1).

#### *1-(6,7-dimethoxy-3,4-dihydroisoquinolin-2(1H)-yl)-2-(4-methoxyphenylamino) ethanone* (8c)

Yield 83 %; white solid, MF: C_20_H_24_N_2_O_4_; MW: 356.17; MP: 116–117 °C; IR (KBr, ν, cm^−1^): 3356, 2951, 2833, 1643, 1517, 1365, 1253, 1211, 1114, 821; ^1^H NMR (400 MHz, CDCl_3_) δ 6.83 (d, *J* = 8.5 Hz, 2H), 6.72 – 6.58 (m, 4H), 4.74 (s, 1H), 4.57 (s, 1H), 3.96 (s, 2H), 3.93 – 3.86 (m, 7H), 3.78 (s, 3H), 3.69 (t, *J* = 5.9 Hz, 1H), 2.89 (t, *J* = 5.6 Hz, 1H), 2.83 (t, *J* = 5.7 Hz, 1H); MS: m/z 257 (M + 1).

#### *1-(6,7-dimethoxy-3,4-dihydroisoquinolin-2(1H)-yl)-2-(3-fluorophenylamino) ethanone* (8d)

Yield 76 %; white solid, MF: C_19_H_21_FN_2_O_3_; MW: 344.15; MP: 144–145 °C; IR (KBr, ν, cm^−1^): 3361, 2981, 2910, 2833, 1656, 1612, 1517 1433, 1365, 1253, 1222, 1112; ^1^H NMR (400 MHz, CDCl3) δ 7.19 – 7.08 (m, 1H), 6.69 – 6.65 (m, 2H), 6.49 – 6.38 (m, 2H), 6.34 (dq, J = 11.5, 2.3 Hz, 1H), 4.75 (s, 1H), 4.57 (s, 1H), 3.96 (d, J = 2.4 Hz, 2H), 3.93 – 3.88 (m, 7H), 3.69 (t, J = 5.9 Hz, 1H), 2.87 (dt, J = 25.9, 5.8 Hz, 2H); MS: m/z 345 (M + 1).

#### *1-(6,7-dimethoxy-3,4-dihydroisoquinolin-2(1H)-yl)-2-(4-fluorophenylamino) ethanone* (8e)

Yield 81 %; white solid, MF: C_19_H_21_FN_2_O_3_; MW: 344.15; MP: 126–12 °C; IR (KBr, ν, cm^−1^): 3365, 2939, 1837, 1643, 1514, 1440, 1265, 1222, 1116; ^1^H NMR (400 MHz, CDCl_3_) δ 6.93 (t, *J* = 8.7 Hz, 2H), 6.69 – 6.64 (m, 2H), 6.61 (ddd, *J* = 8.9, 4.3, 2.1 Hz, 2H), 4.85 (s, 1H), 4.74 (s, 1H), 4.57 (s, 1H), 3.95 (s, 2H), 3.91 – 3.85 (m, 7H), 3.69 (t, *J* = 5.9 Hz, 1H), 2.90 (t, *J* = 5.8 Hz, 1H), 2.83 (t, *J* = 5.9 Hz, 1H); MS: m/z 345 (M + 1).

#### *2-(2-chlorophenylamino)-1-(6,7-dimethoxy-3,4-dihydroisoquinolin-2(1H)-yl) ethanone* (8f)

Yield 68 %; white solid, MF: C_19_H_21_ClN_2_O_3_; MW: 360; MP: 104–105 °C; IR (KBr, ν, cm^−1^): 3379, 2999, 2933, 2835, 1665, 1517, 1485, 1415, 1257, 1228, 1209, 1111; ^1^H NMR (400 MHz, CDCl_3_) δ 7.31 (dd, *J* = 7.9, 1.4 Hz, 1H), 7.17 (t, *J* = 7.7 Hz, 1H), 6.68 (s, 2H), 6.66 – 6.58 (m, 2H), 4.76 (s, 1H), 4.59 (s, 1H), 4.03 (d, *J* = 1.9 Hz, 2H), 3.93 (s, 1H), 3.89 (d, *J* = 3.2 Hz, 6H), 3.70 (t, *J* = 5.9 Hz, 1H), 2.91 (t, *J* = 5.7 Hz, 1H), 2.85 (t, *J* = 5.9 Hz, 1H); MS: m/z 361 (M + 1).

#### *2-(3-chlorophenylamino)-1-(6,7-dimethoxy-3,4-dihydroisoquinolin-2(1H)-yl)ethanone* (8g)

Yield 84 %; white solid, MF: C_19_H_21_ClN_2_O_3_; MW: 360; MP: 136–137 °C; IR (KBr, ν, cm^−1^): 3367, 3053, 2954, 2933, 2833, 1656, 1593, 1514, 1392, 1261, 1228, 1116; ^1^H NMR (400 MHz, CDCl_3_) δ 7.14 – 7.06 (m, 1H), 6.71 (dd, *J* = 7.9, 1.2 Hz, 1H), 6.67 (d, *J* = 3.2 Hz, 2H), 6.63 – 6.60 (m, 1H), 6.56 (dt, *J* = 8.2, 2.2 Hz, 1H), 4.73 (d, *J* = 15.0 Hz, 1H), 4.57 (s, 1H), 3.97 – 3.92 (m, 2H), 3.89 (dd, *J* = 8.1, 5.4 Hz, 7H), 3.68 (t, *J* = 5.9 Hz, 1H), 2.90 (t, *J* = 5.9 Hz, 1H), 2.84 (t, *J* = 5.8 Hz, 1H); MS: m/z 361 (M + 1).

#### *2-(4-chlorophenylamino)-1-(6,7-dimethoxy-3,4-dihydroisoquinolin-2(1H)-yl) ethanone* (8h)

Yield 75 %; white solid, MF: C_19_H_21_ClN_2_O_3_; MW: 360; MP: 115–116 °C; IR (KBr, ν, cm^−1^): 3360, 2927, 2906, 2833, 1658, 1600, 1511, 1433, 1265, 1226, 1124; ^1^H NMR (400 MHz, CDCl_3_) δ 7.16 (d, *J* = 8.7 Hz, 2H), 6.69 – 6.63 (m, 2H), 6.59 (dd, *J* = 8.9, 2.3 Hz, 2H), 4.74 (s, 1H), 4.57 (s, 1H), 3.95 (d, *J* = 2.9 Hz, 2H), 3.92 – 3.87 (m, 7H), 3.68 (s, 1H), 2.90 (t, *J* = 5.9 Hz, 1H), 2.84 (s, 1H); MS: m/z 361 (M + 1).

#### 2-(2-bromophenylamino)-1-(6,7-dimethoxy-3,4-dihydroisoquinolin-2(1H)-yl) ethanone (8i)

Yield 68 %; white solid, MF: C_19_H_21_BrN_2_O_3_; MW: 404.07; MP: 109–110 °C; IR (KBr, ν, cm^−1^): 3379, 2999, 2931, 2837, 1649, 1517, 1433, 1442, 1257, 1228, 1207; ^1^H NMR (400 MHz, CDCl_3_) δ 7.50 (dd, *J* = 9.4, 4.5 Hz, 1H), 7.22 (s, 1H), 6.69 – 6.64 (m, 2H), 6.64 – 6.53 (m, 2H), 4.76 (s, 1H), 4.58 (s, 1H), 4.06 (s, 1H), 3.93 (t, *J* = 5.9 Hz, 1H), 3.91 – 3.83 (m, 7H), 3.70 (t, *J* = 5.9 Hz, 1H), 2.91 (t, *J* = 5.7 Hz, 1H), 2.85 (t, *J* = 5.7 Hz, 1H); MS: m/z 405 (M + 1).

#### *2-(3-bromophenylamino)-1-(6,7-dimethoxy-3,4-dihydroisoquinolin-2(1H)-yl)ethanone* (8j)

Yield 75 %; white solid, MF: C_19_H_21_BrN_2_O_3_; MW: 404.07; MP: 123–124 °C; IR (KBr, ν, cm^−1^): 3369, 2954, 2931, 2833, 1649, 1591, 1514, 1485, 1433, 1259, 1228, 1112; ^1^H NMR (400 MHz, CDCl3) δ 7.06 (t, J = 8.0 Hz, 1H), 6.86 (dd, J = 7.8, 1.0 Hz, 1H), 6.77 (dd, J = 4.0, 2.0 Hz, 1H), 6.67 (d, J = 2.7 Hz, 2H), 6.61 (dd, J = 8.2, 2.3 Hz, 1H), 4.75 (s, 1H), 4.57 (s, 1H), 3.95 (d, J = 1.2 Hz, 2H), 3.92 (s, 2H), 3.91 – 3.88 (m, 5H), 3.69 (t, J = 5.9 Hz, 1H), 2.91 (t, J = 5.8 Hz, 1H), 2.84 (t, J = 5.9 Hz, 1H); MS: m/z 405 (M + 1).

#### 2-(4-bromophenylamino)-1-(6,7-dimethoxy-3,4-dihydroisoquinolin-2(1H)-yl)ethanone (8k)

Yield 72 %; white solid, MF: C_19_H_21_BrN_2_O_3_; MW: 404.07; MP: 117–118 °C; IR (KBr, ν, cm^−1^): 3358, 2985, 2902, 2833, 1643, 1517, 1433, 1396, 1285, 1224, 1124; ^1^H NMR (400 MHz, CDCl_3_) δ 7.31 (s, 1H), 6.67 (d, *J* = 5.2 Hz, 2H), 6.64 – 6.45 (m, 3H), 5.02 (s, 1H), 4.74 (s, 1H), 4.57 (s, 1H), 3.94 (s, 2H), 3.91 – 3.87 (m, 7H), 3.68 (t, *J* = 5.8 Hz, 1H), 2.90 (t, *J* = 5.8 Hz, 1H), 2.84 (t, *J* = 6.0 Hz, 1H); MS: m/z 405 (M + 1).

#### 4-(2-(6,7-dimethoxy-3,4-dihydroisoquinolin-2(1H)-yl)-2-oxoethylamino)benzonitrile (8l)

Yield 68 %; white solid, MF: C_20_H_21_N_3_O_3_; MW: 351.16; MP: 146–147 °C; IR (KBr, ν, cm^−1^): 3358, 2985, 2902, 2833, 2208, 1643, 1517, 1433, 1396, 1285, 1224, 1124; ^1^H NMR (400 MHz, CDCl_3_) δ 7.48 (d, *J* = 8.5 Hz, 2H), 6.68 (d, *J* = 4.6 Hz, 2H), 6.65 – 6.62 (m, 2H), 5.60 (s, 1H), 4.75 (s, 1H), 4.57 (s, 1H), 4.00 (t, *J* = 4.0 Hz, 2H), 3.91 (s, 1H), 3.89 (t, *J* = 2.7 Hz, 6H), 3.68 (s, 1H), 2.91 (t, *J* = 5.7 Hz, 1H), 2.85 (t, *J* = 5.7 Hz, 1H); MS: m/z 352 (M + 1).

#### 2-(3-acetylphenylamino)-1-(6,7-dimethoxy-3,4-dihydroisoquinolin-2(1H)-yl)ethanone (8m)

Yield 66 %; white solid, MF: C_21_H_24_N_2_O_4_; MW: 368.17; MP: 111–112 °C; IR (KBr, ν, cm^−1^): 3365, 2937, 2835, 1672, 1643, 1598, 1517, 1359, 1315, 1259, 1224, 1116; ^1^H NMR (400 MHz, CDCl_3_) δ 7.36 – 7.30 (m, 2H), 7.22 – 7.20 (m, 1H), 6.94 – 6.90 (m, 1H), 6.68 (s, 2H), 4.75 (s, 1H), 4.60 (s, 1H), 4.03 (s, 2H), 3.94 (s, 1H), 3.90 – 3.88 (m, 6H), 3.71 (t, *J* = 5.9 Hz, 1H), 2.91 (t, *J* = 5.8 Hz, 1H), 2.84 (t, *J* = 5.9 Hz, 1H), 2.61 (s, 3H); MS: m/z 369 (M + 1).

#### (6,7-dimethoxy-3,4-dihydroisoquinolin-2(1H)-yl)-2-(3(trifluoromethyl)phenylamino) ethanone (8n)

Yield 76 %; white solid, MF: C_20_H_21_F_3_N_2_O_3_; MW: 394.15; MP: 135–136 °C; IR (KBr, ν, cm^−1^): 3350, 2910, 2835, 1643, 1612, 1514, 1431, 1359, 1284, 1261, 1222, 1109; ^1^H NMR (400 MHz, CDCl_3_) δ 7.30 (s, 1H), 6.99 (d, *J* = 7.7 Hz, 1H), 6.84 (s, 2H), 6.68 (t, *J* = 3.1 Hz, 2H), 4.76 (s, 1H), 4.59 (s, 1H), 4.00 (s, 2H), 3.92 (s, 1H), 3.90 (d, *J* = 2.5 Hz, 6H), 3.71 (t, *J* = 5.9 Hz, 1H), 2.92 (t, *J* = 5.9 Hz, 1H), 2.85 (t, *J* = 5.8 Hz, 1H); MS: m/z 395 (M + 1).

#### 1-(6,7-dimethoxy-3,4-dihydroisoquinolin-2(1H)-yl)-2-(2,5-dimethylphenylamino) ethanone (8o)

Yield 75 %; white solid, MF: C_21_H_26_N_2_O_3_; MW: 354.19; MP: 140–141 °C; IR (KBr, ν, cm^−1^): 3402, 2995, 2833, 1643, 1517, 1415, 1255, 1232, 1207, 1112; ^1^H NMR (400 MHz, CDCl3) δ 6.67 (d, J = 4.6 Hz, 2H), 6.44 (s, 1H), 6.35 (s, 2H), 4.74 (s, 1H), 4.58 (s, 1H), 3.98 (s, 2H), 3.91 (s, 1H), 3.89 (t, J = 4.3 Hz, 6H), 3.70 (t, J = 5.9 Hz, 1H), 2.90 (t, J = 5.9 Hz, 1H), 2.83 (t, J = 6.0 Hz, 1H), 2.28 (s, 6H); MS: m/z 355 (M + 1).

## Conclusion

In summary, we designed and synthesized novel series of 2-(6,7-dimethoxy-3,4-dihydroisoquinolin-2(*1H*)-yl)-*N*-phenylacetamide and 1-(6,7-dimethoxy-3,4-dihydroisoquinolin-2(*1H*)-yl)-2-(phenylamino)ethanone as HIV-1 RT inhibitors. *In-vitro* screening of the synthesized compounds against HIV-1 RT exhibited weak to significant inhibitory activity. Compounds of series **8** exhibited more significant HIV-1 RT inhibition as compared to the compounds of **5** series. Among the tested compounds, eighteen compounds (**5d, 5f, 5h, 5n, 5o, 8b, 8c, 8e, 8f, 8g, 8h, 8i, 8j, 8k, 8l, 8m, 8n** and **8o**) exhibited more than 50 % inhibition at tested 100 μM concentration and two compounds **8h** and **8l** showed promising inhibition (74.82 and 72.58 %) respectively. SAR analysis of compounds **5a-o** demonstrated that, substitution of phenyl ring with electron donating group at *para* position and electron withdrawing especially chloro at *ortho* position resulted in enhanced potency. Among the compounds **8a-o**, substitution with electron withdrawing group of moderate to large size at *para* position of phenyl ring significantly increased the potency against HIV-1 RT. Docked poses of compounds **8h** and **8l** in the NNIBP of RT showed strong hydrophobic and hydrophilic interaction, may be responsible for their significant *in-vitro* activity. Predicted physiochemical parameters of the synthesized compounds were within the acceptable range of drugable properties. Thus overall SAR studies can help in identification of further lead as well as in the designing of newer potential inhibitor of HIV-1 RT.

## Abbreviations

ADMET: Absorption, distribution, metabolism, excretion and toxicity
HBA: Hydrogen bond acceptors
HBD: Hydrogen bond donors
MW: Molecular weight
TLC: Thin Layer Chromatography
